# A Review of the Risk Factors for Iron Deficiency Anaemia among Adolescents in Developing Countries

**DOI:** 10.1155/2023/6406286

**Published:** 2023-01-03

**Authors:** Michael Akenteng Wiafe, Jessica Ayenu, Divine Eli-Cophie

**Affiliations:** ^1^Department of Nutritional Sciences, School of Allied Health Sciences, University for Development Studies, Tamale, Ghana; ^2^Department of Clinical Nutrition and Dietetics, School of Allied Health Sciences, College of Health and Allied Sciences, University of Cape Coast, Cape Coast, Ghana; ^3^Department of Sport Nutrition, University of Health and Allied Sciences, Ho, Ghana

## Abstract

**Introduction:**

Identifying the root causes of iron deficiency anaemia is a prerequisite for effective management and prevention in adolescents. This systematic review assessed risk factors of iron deficiency anaemia among adolescents living in developing countries.

**Method:**

Electronic databases such as PubMed, Cochrane Library, Science Direct, Google Scholar, and SCOPUS were comprehensively searched for studies published between 1990 and 2020 that involved risk factors of iron deficiency anaemia among adolescents living in developing countries. The quality of the included studies was assessed using the American Dietetic Association Quality Criteria Checklist.

**Results:**

A total of 2,252 publications were reviewed, and only fifteen cross-sectional studies were eligible for inclusion, eight of which focused on female adolescents and seven on both genders. Direct risk factors contributing to anaemia among adolescents included food intake practices (*n* = 10 studies), female adolescents (*n* = 8 studies), menstruation (*n* = 5 studies), and parasitic infection (*n* = 6 studies). Indirect risk factors found to be associated with anaemia among adolescents included low educational status (*n* = 4 studies) and low socioeconomic status (*n* = 3 studies). All fifteen studies were of good quality.

**Conclusion:**

Food intake practices, female adolescents, menstruation, parasitic infection, and low educational status were the leading risk factors of iron deficiency anaemia among adolescents. Further research should concentrate on assessing the effectiveness and efficacy of existing interventions aimed at preventing iron deficiency among vulnerable groups in developing countries.

## 1. Introduction

Adolescents undergo physiological and psychological growth to set the foundation of adulthood. The biological well-being of adolescents requires improved nourishment. It has been revealed that prolonged insufficient intake of foods rich in micronutrients such as iron, zinc, and vitamin A relevant to support the biological metamorphosis in adolescents can adversely affect their growth and well-being [1]. The majority of adolescents habitually skip breakfast, fruits, vegetables, and milk daily, reducing their dietary intake [2, 3]. Adolescents with such dietary practices manifest micronutrient inadequacies such as iron, calcium, zinc, folic acid, and vitamins A, D, and C [4, 5]. These deficiencies expose adolescents to perpetual nutritional and health vulnerabilities.

Deficiencies of iron, folate, and vitamin B_12_ contribute to nutritional anaemia in adolescents [6, 7]. Among the different types of nutritional anaemias, iron deficiency anaemia is the most prevalent [8–10]. Iron deficiency anaemia (IDA) is measured with indicators such as haemoglobin, serum ferritin, transferrin receptors, transferrin saturation/total iron binding capacity, and zinc protoporphyrin [11, 12]. Anaemia is mostly defined as low haemoglobin levels in the blood or haemoglobin levels less than 120 g/l in adolescents [13]. 

Statistics show that about 30–35% of the world's population suffers from iron deficiency anaemia, which affects about 47.5% of people living in Africa [7, 14]. Populations most at risk of iron deficiency anaemia are children aged less than five years, adolescents, women of reproductive age, pregnant women, and lactating mothers [14].

To ameliorate the prevalence and consequences of iron deficiency anaemia in adolescents, a review study recommended the identification of localized risk factors of iron deficiency anaemia to aid in effective management and prevention [15]. Predictors of iron deficiency anaemia among adolescents have been reported by different studies in several countries [16–18]. In developing countries, risk factors of IDA include but are not limited to malaria, worm infestation, low dietary iron intake, micronutrient deficiencies, the human immunodeficiency virus, and inherited disorders [19]. The wide variety of contributory factors of IDA reported by many studies negatively impacts adolescent's health. Due to this, several interventions have failed to reduce the high prevalence of IDA among adolescents in the long term.

Iron deficiency anaemia negatively impacts the educational and economic well-being of adolescents. It has been associated with stunting, wasting, being underweight, poor cognitive function, low physical activity, and attention deficit hyperactive disorders in adolescents [20–26]. Iron deficiency anaemia is now known to be the leading cause of disability adjusted live years in adolescents [27].

To successfully address iron deficiency anaemia, it is critical to holistically identify the key risk factors affecting adolescents that contribute to this deficiency. The systematic review assessed the risk factors of iron deficiency anaemia among adolescents living in developing countries.

## 2. Method

### 2.1. Study Design and Search Strategy

This systematic review was conducted following the guidelines provided by Preferred Reporting Items for Systematic Reviews and Meta-Analyses (PRISMA) [28]. The current review defined an adolescent as an individual with an age ranging from 10 to 19 years [29]. The list of developing countries considered was based on country classifications by the United Nations Children's Fund [30]. A comprehensive search of articles published from January 1990 to December 2020 was sourced from Google Scholar, PubMed, Scopus, Science direct, and Cochrane Library. The search terms used either singly or in combination included risk factors of anaemia, iron deficiency anaemia, determinants of anaemia, predictors of anaemia, anaemia in adolescents, low haemoglobin, anaemia, and adolescents.

### 2.2. Study Selection and Eligibility Criteria

The review focused on studies with the primary objective of identifying risk factors of iron deficiency anaemia (IDA) among adolescents living in developing countries. Studies conducted among adolescents without any underlying health conditions were included. Research conducted among adolescents with pregnancy, lactating, sickle cell, or genetic haemoglobin or with any underlying condition, age group mixed, and other forms of anaemia were excluded. Studies with inaccessible full articles, non-English written articles, and articles published before 1990 were also excluded.

### 2.3. Outcomes Assessed

The outcomes assessed in this review were risk factors of iron deficiency anaemia among adolescents. The primary outcomes were the risk factors directly associated with adolescents contributing to iron deficiency anaemia. The secondary outcomes were risk factors indirectly associated with adolescents, leading to iron deficiency anaemia.

### 2.4. Data Extraction and Synthesis

The primary information from each study was extracted by one researcher. The primary information for the review included the following: author (s), country, year, gender, age, sample size, and risk factors of iron deficiency anaemia. The data are summarized in Table 1.

### 2.5. Quality Assessment

The methodological quality assessment of each study was checked using the American Dietetic Association Quality Criteria Checklist [46]. The overall quality of each study was rated positive, negative, or neutral. The findings of the quality assessments are shown in Table 2.

## 3. Result

### 3.1. Study Selection

**Figure 1:**
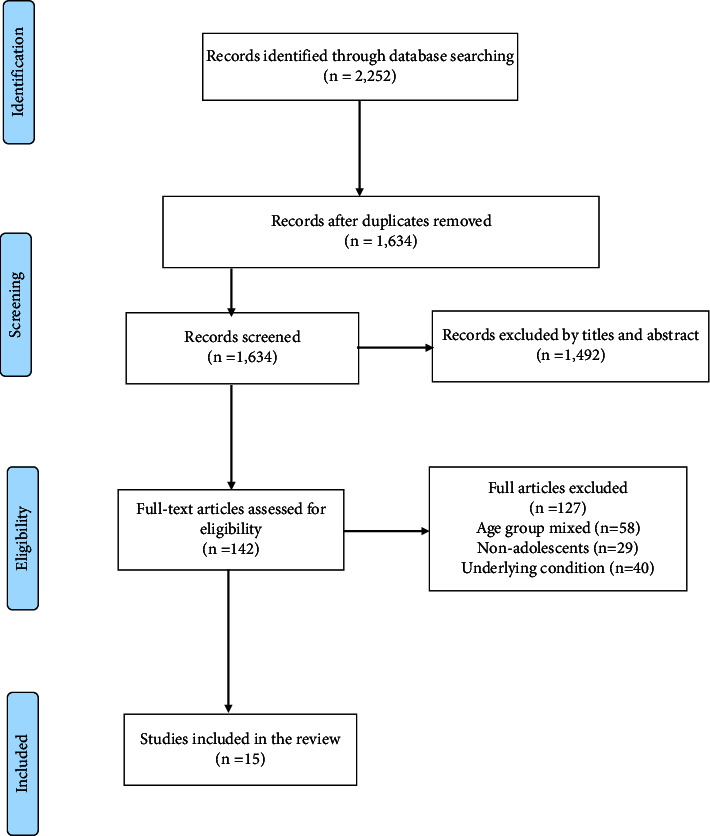
PRISMA diagram of the review.

A total of 2,252 articles were retrieved from the five databases. Six hundred and eighteen (618) duplicates were removed and one thousand, four hundred and ninety-two (1,492) records were excluded after screening by title and abstract. One hundred and forty-two (142) articles were fully assessed for eligibility, and one hundred and twenty-seven (127) articles were also excluded based on the inclusion and exclusion criteria. Finally, fifteen (15) studies were included in the review (Figure 1).

### 3.2. Study Characteristics

All studies included in this review were cross-sectional and published between the years 1990 and 2020. Five studies were conducted in Ethiopia [31, 32, 34, 36, 38]; four studies were also conducted in India [37, 39, 40, 43]; two studies were conducted in Kenya [41, 44]; and one study was conducted in Ghana [33], Egypt [45], Nepal [35], and Iran [42]. Only two of the studies had nationwide representation [35, 45]. Eight of the studies recruited exclusively female adolescents and seven of the studies recruited adolescents of mixed gender. The studies had sample sizes ranging from 137 to 2,032. Participants were aged 10–19 years (Table 1).

### 3.3. Assessment of the Study Quality

Table 2 shows the quality assessment of the studies using the quality control checklist. All studies were cross-sectional, and attributes of the quality criteria checklist were modified for maximum use in assessment. Attributes such as “comparable study groups” and “use of blinding” were not applicable for the assessment of the studies. All fifteen studies had a positive quality rating.

### 3.4. Associated Factors of Adolescent Iron Deficiency Anaemia

**Figure 2:**
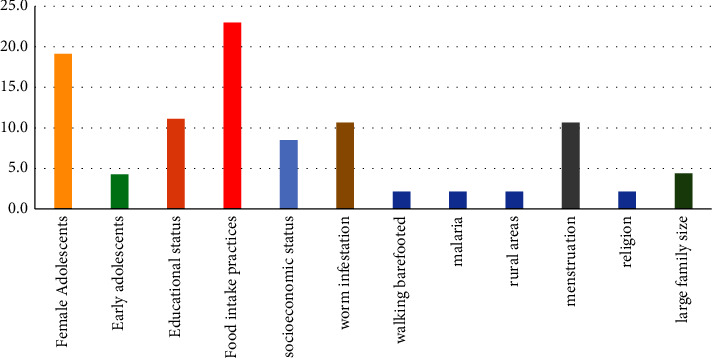
Risk factors of anaemia. Education: low maternal education^1^ and adolescent; food intake practices: vegetarian diet^1^, low dietary iron intake, lower meal frequency, lower dietary diversity, meal skipping, and snacking; menstruation: excessive bleeding^1^ and menarche. ^1^ indicates majority.

The various studies assessed different factors contributing to iron deficiency anaemia among adolescents in developing countries. For this review, the factors have been grouped under direct factors: food intake practices, malaria infection, worm infestation, female adolescents, blood loss, and indirect factors: educational status, socioeconomic status, rural areas, family size, religion, and walking barefoot (Figure 2).

### 3.5. Food Intake Practices

Ten studies assessed the relationship between iron deficiency anaemia and food intake practices among adolescents. Three of these studies indicated that vegetarian dietary practices increased the odds of anaemia among adolescents [39, 40, 43]. Kuar et al. [42] reported that vegetarian adolescents had higher odds of being anaemic than those who consumed a mixed diet (OR = 8.5, 95% CI = 5.7–12.8). In another cross-sectional study, vegetarian adolescents had a 4.4% greater chance of being anaemic than their counterparts who did not practice vegetarianism [40]. Two studies reported on dietary diversity. The studies documented that low dietary diversity significantly increased adolescents' risk (AOR = 3.57, 95% CI: 1.88–6.75) [31] and (AOR = 2.33, 95% CI: 1.2–4.86) [32] of being anaemic. Furthermore, low dietary iron intake significantly increased anaemia among adolescents [41, 43]. Two studies reported on meal skipping and meal frequency [33, 36]. Adolescents who skipped meals and had a low meal frequency had high chances of being anaemic. One study among adolescents in Ghana showed that snacking was not significantly associated with anaemia (*β* = 0.484, *p* > 0.05) [33].

### 3.6. Parasitic Infections

Six studies reported on parasitic infections. Malaria and worm infestations, particularly schistosomiasis and ova ascaris, were the common parasitic infections found among adolescent girls in the studies included in this review. Five studies indicated that worm infestation significantly increased the odds of anaemia incidence in adolescents [40–44]. One cross-sectional study reported that malaria significantly increased the odds of anaemia in adolescents (OR = 3.68, 95% CI: 1.69–7.98) [41].

### 3.7. Female Adolescents

Eight studies reported on the relationship between female adolescents and anaemia [31, 32, 35, 37–41]. The studies showed significant and higher odds of female adolescents having anaemia. Four out of eight studies showed that older adolescents had higher odds of anaemia [35, 37, 38, 41].

### 3.8. Blood Loss/Menstruation

Five studies [31, 38, 42–44] assessed the relationship between anaemia and menstruation. Three out of five studies indicated that excessive bleeding during menstruation significantly increased the odds of anaemia [31, 43, 44]. According to Fentie et al. [31], adolescent girls who bleed for more than 5 days have an increased risk (AOR = 2.25, 95% CI: 1.17–4.33) of being anaemic. Regasa and Haidar [38] reported menstruation to be a statistically significant risk factor for anaemia in adolescents. However, Ramzi et al. [42] found no significant association between menstruation and anaemia in adolescents.

### 3.9. Educational Status

Among the four studies that reported on educational status as a risk factor of anaemia, three studies indicated that maternal education was a key determinant [32, 39, 45]. El Sahn et al. [45]; reported that the risk of anaemia increased significantly with decreased level of education (OR = 3.5, 95% CI: 10.90–6.32). Adolescents with education up to the junior high school level or lower were found to have increased odds of being anaemic [32, 33].

### 3.10. Socioeconomic Status

Three studies assessed the relationship between socioeconomic status and anaemia. The outcome indicated that low socioeconomic status increased the odds of anaemia in adolescents [31, 39, 45]. All studies found adolescents with low socioeconomic backgrounds to have increased odds of being anaemic, OR = 2.16, 95% CI: 1.17–4.33; OR 2.86, 95% CI: 1.16–7.04; OR = 1.4, 95% CI: 1.13–1.8; Fentie et al. [31]; Agrawal et al. [39]; and El Sahn et al. [45], respectively.

### 3.11. Rural Areas

Two studies reported on rural areas and anaemia [36, 38]. The studies showed that adolescents living in rural areas had an increased risk of anaemia. Regasa and Haidar found that the odds were statistically significant.

### 3.12. Family Size

Two studies assessed the relationship between family size and anaemia [36, 42]. The studies indicated that large family size increased the odds of anaemia. Ramzi et al. [42] showed significant association, while Shaka and Wondimagegne [36] indicated otherwise.

### 3.13. Religion

Only one study investigated the association between religion and anaemia. However, the results of the study showed no significant effects of religion on anaemia among adolescents [39].

### 3.14. Walking Barefooted

Only one study reported that adolescents who walk barefooted have higher odds of having anaemia [35]. According to the authors, adolescents who walked barefoot had a 1.78 chance of being anaemic (AOR = 1.78, 95% CI: 1.08, 2.94).

## 4. Discussion

The prevalence of anaemia among adolescents is of public health concern despite the application of varied interventions. Management and prevention of iron deficiency anaemia are complex, indicating that different factors contribute to IDA in different geographical settings. The present study assessed the risk of iron deficiency anaemia among adolescents in developing countries. The risk factors of iron deficiency anaemia among adolescents are conglomerate. However, food intake practices, low educational status, parasitic infections, older adolescent girls, menstruation, and low socioeconomic status were the leading risk factors that predispose adolescents to iron deficiency anaemia (Figure 2).

### 4.1. Food Intake Practices

Adolescents prefer to explore their dietary environment and, thus, consume foods that are pleasing to the eyes with little or no consideration of the nutrients needed for their growth and well-being. Most adolescents binge on junk foods due to the neglect of a nutritious diet [47]. These poor food choices affect their nutrient needs, leading to micronutrient deficiencies, particularly anaemia. The negative effects of IDA on learning, scholastic performance, and achievement among adolescents contribute to dropout rates [21, 23, 25]. Adolescents with low educational status are unable to gain employable skills, thereby affecting their economic status [48]. Unskilled labour pays less as guardians are unable to give their children a good education and also meet their nutritional needs. These adolescents also become mothers of children with iron deficiency anaemia to perpetuate the cycle of consequences of anaemia. In this review, most of the studies found that vegetarian dietary practices increased the risk of anaemia among adolescents [39, 40, 43]. Inadequate dietary iron intake [41, 43] and low dietary diversity [31, 32] were second in contributing to IDA among adolescents. Most iron-rich food sources are expensive in developing countries [49]. Other food intake practices, such as meal skipping, lower meal frequency, lower dietary diversity, household food insecurity, and snacking, also increased the risk of IDA among adolescents. Poor nutrition has been a major risk factor for IDA among adolescents [40, 50].

### 4.2. Adolescent Girls

Our review showed that female adolescents had a higher risk of iron deficiency anaemia, particularly older girls. It was thus not surprising that eight of the fifteen studies focused on female adolescents [31, 34, 37, 38, 41–44]. Older girls may prefer to eat out of home, skip meals, and diet to maintain certain body curvature, making them more vulnerable to IDA. Most guardians have less control over an older adolescent girl's food intake. The fear of gaining weight and low nutrition knowledge influence the eating habits of adolescents and contribute to IDA [51, 52]. Menstruation and childbearing have increased the odds of anaemia in older adolescents. A nationwide study in Namibia, Malawi, Zimbabwe, and Mozambique showed that anaemic mothers have higher odds of delivering children with low haemoglobin levels [53].

### 4.3. Worm Infestation

The prevalence of worm infestations is estimated to be about 1.5 billion, with the majority of the population from sub-Saharan Africa, the Americas, China, and East Asia [54]. Roundworm (*Ascaris lumbricoides*), whipworm (Trichuris trichiura), hookworm (Necator americanus and Ancylostoma duodenale), and other helminths have been implicated in anaemia by causing gastrointestinal blood loss, poor nutrient absorption, inhibition or suppression of appetite, and general inflammation among adolescents [40, 54–56].

### 4.4. Guardian Education

Low educational status of guardians, particularly mothers, has been linked to a high risk of anaemia in adolescents in diverse settings and studies [32, 39, 57]. Mothers with limited formal education may not be able to read and understand food labels. Knowledge levels of nutrition by mothers are critical as most are key kitchen persons in most homes influencing food preparation, dietary choices, and intake of the family. Maternal education status has been shown to influence children's normal haemoglobin levels [58]. The education level of fathers and adolescents rarely led to iron deficiency anaemia within our target group.

### 4.5. Socioeconomic Status

Higher maternal education and employment status reduce the odds of iron deficiency anaemia in children [59]. Guardians with low educational status have low skilled employment with poor remuneration [60]. Low socioeconomic status due to unemployment affects the purchasing power of the household. Adolescents largely depend on guardians or parents for their financial and dietary needs. Households with low socioeconomic status face the risk of food insecurity, low dietary diversity, and inadequate food intake, which pose health risks [61, 62].

### 4.6. Strength and Limitations

The study gives an overview of the risk factors of iron deficiency anaemia among adolescents in developing countries. The sample sizes of most of the studies were not nationally representative, and female adolescents were the target for most of the studies; therefore, the outcome cannot be generalized.

## 5. Conclusion and Further Directions

The review showed that food intake practices, parasitic infections, menstruation, increasing age of female adolescents, and low educational status of guardians were the leading risk factors of iron deficiency anaemia among adolescents in most developing countries. Funding agencies should support nationally representative nutrition research to continue to identify localized risk factors that precipitate IDA among adolescents. Further studies should focus on assessing the effectiveness and efficacy of already existing interventions such as iron-folic acid supplementation, nutrition education, use of insecticide mosquito nets, and intermittent deworming of adolescents, and developing appropriate policies and programmes to strenghten such interventions. Developing countries should continue to adopt policies and programmes to sustain girl child education, maternal education, and economic empowerment of guardians, particularly women, to reduce the prevalence and menace of IDA in adolescents. Governments and non-governmental organizations should prioritize adolescent nutrition as it is another gateway to having a positive impact on the lifecycle.

## Figures and Tables

**Table 1:** Summary of studies included in the systematic review.

Study details	Year	Study design	Gender and age (years)	Sample size	Summary of results
Fentie et al. [31]; Ethiopia	2019	Cross-sectional	Girls; 14–19	528	Adolescent girls living alone (AOR = 4.430, 95% CI = 2.20–8.90)^*∗*^, low dietary diversity score (AOR = 3.57, 95% CI = 1.88–6.75)^*∗*^, excessive menstrual bleeding (AOR = 2.25, 95% CI = 1.17–4.33)^*∗*^, and low economic status (AOR = 2.16, 95% CI = 1.17–4.33)^*∗*^ were positively associated with anaemia
Fage et al. [32]; Ethiopia	2017	Cross-sectional	Both; 10–19	493	Female adolescent (AOR = 2.31, 95% CI = 1.51–3.54)^*∗*^, low educational status of adolescent (AOR = 1.66, 95% CI = 1.004–2.77)^*∗*^, illiterate mothers (AOR = 2.23, 95% CI = 1.02–4.89)^*∗*^, and low dietary diversity score (AOR = 2.33, 95% CI = 1.12–4.86)^*∗*^ increased odds of anaemia
Wiafe et al. [33]; Ghana	2019	Cross-sectional	Both; 10–14	137	Meal skipping (OR = 1.4, 95% CI = 0.7–3.0), snacking (OR = 1.6, 95% CI = 0.7–3.6), and adolescent with JHS education (OR = 1.7, 95% CI = 0.7–4.0) were positively associated with anaemia
Gebreyesus et al. [34]; Ethiopia	2015	Cross-sectional	Girls; 10–19	1323	Early adolescents (AOR = 1.98, 95% CI = 1.03–3.82)^*∗*^ and food insecure household (AOR = 1.48, 95% CI = 1.05–2.049)^*∗*^ increased the risk of anaemia
Chalise et al. [35]; Nepal	2014	Cross-sectional	Both; 10–19	3780	Older adolescents (AOR = 1.75, 95% CI = 1.44–2.13)^*∗*^, female adolescents (AOR = 2.02, 95% CI = 1.57–2.60)^*∗*^, and walking barefooted (AOR = 1.78, 95% CI = 1.08–2.94) increased risk of anaemia
Shaka and Wondimagegne [36]; Ethiopia	2016	Cross-sectional	Both; 10–19	443	Early adolescents (AOR = 4.75, 95% CI = 1.69–13.35)^*∗*^, large family size (AOR = 9.82, 95% CI = 2.42–39.88), adolescents living in rural areas (AOR = 4.37, 95% CI = 1.54–12.46), and lower meal frequency (AOR = 3.25, 95% CI = 1.42–7.45) increased the odds of anaemia
Ahankari et al. [37]; India	2014–2015	Cross-sectional	Girls; 13–17	1,010	Anaemia was associated with older adolescents (AOR = 1.41, 95% CI = 1.17–1.70)^*∗*^
Regasa and Haidar [38]; Ethiopia	2016	Cross-sectional	Girls; 10–19	448	Late adolescent (AOR = 3.8, 95% CI = 2.3–8.5)^*∗*^, adolescents living in rural areas (AOR = 3.4 95% CI = 1.9–7.0)^*∗*^, and menarche (AOR = 2.3 95% CI = 1.34–4.2)^*∗*^ increased odds of anaemia
Agrawal et al. [39]; India	2014–2015	Cross-sectional	Both; 10–19	526	Religion (Muslim) (AOR = 1.4, 95% CI = 0.82–2.43), female gender (AOR = 1.9, 95% CI = 1.3–2.7)^*∗*^, illiterate mothers (AOR = 1.42, 95% CI = 0.62–3.24), vegetarian diet (AOR = 2.28, 95% CI = 0.83–6.22), and occupation (student) (AOR = 2.86, 95% CI = 1.16–7.04)^*∗*^ increased risk of anaemia
Thomas et al. [40]; India	2011–2013	Cross-sectional	Both; 10–18	200	Female gender (OR = 1.70, 95% CI = 0.84–3.43), vegetarian diet (OR = 4.41, 95% CI = 2.04–9.51)^*∗*^, and history of worm infestation (OR = 2.08, 95% CI = 0.96–4.50) ^*∗*^ contributed to anaemia
Nelima [41], Kenya	2015	Cross-sectional	Girls; 14–18	230	Inadequate iron intake (OR = 10.3, 95% CI = 5.2–20.37)^*∗*^, late adolescents (OR = 2.69, 95% CI = 1.46–4.96)^*∗*^, malaria infections (OR = 5.38, 95% CI = 2.84–10.19)^*∗*^, and parasitic infections (OR = 11.94, 95% CI = 2.71–52.57)^*∗*^ were positively associated with anaemia
Ramzi et al. [42]; Iran	2011	Cross-sectional	Girls; 10–19	363	Parasitic infections (OR = 6.83, 95% CI = 1.66–28.11)^*∗*^, large family size (OR = 2.25, 95% CI = 0.91–5.52)^*∗*^, and longer duration of menstruation (OR = 1.78, 95% CI = 0.64–4.93) were associated with anaemia
Kaur et al. [43]; India	2000–2002	Cross-sectional	Girls; 13–19	630	Vegetarian diet (OR = 5.83, 95% CI = 3.73–9.13)^*∗*^, excessive menstrual bleeding (OR = 5.65, 95% CI = 1.26–25.38)^*∗*^, low iron intake (OR = 4.16, CI = 2.08–8.31)^*∗*^, and history of worm infestation (OR = 4.11, CI = 1.70–9.93)^*∗*^ increased odds of anaemia
Leenstra et al. [44]; Kenya	1998–1999	Cross-sectional	Girls; 12–18	648	Heavy menstrual bleeding (OR = 4.29, 95% CI = 1.46–12.64)^*∗*^ and parasitic infections (OR = 2.01 95% CI = 1.02–3.98)^*∗*^ were positively associated with anaemia
El Sahn et al. [45]; Egypt	1997	Cross-sectional	Both; 10–19	1,980	Low educational status (OR = 3.46, 95% CI = 1.90–6.32)^*∗*^, low socioeconomic status (OR = 1.43, 95% CI = 1.13–1.81)^*∗*^, and increased risk of anaemia

^*∗*^ *p* value is significant.

**Table 2:** Quality assessment of the studies using quality control checklist.

Study	Clear research question	Participant selection free from bias	Comparable study groups	Participant withdrawals or response rate described	Use of blinding	Description of intervention protocol and/or data collection procedures	Outcomes clearly defined	Appropriate statistical analysis	Conclusions supported by results	Unlikely funding bias	Overall quality rating
Fentie et al. [31]	+	+	N/A	+	N/A	+	+	+	+	+	+
Fage et al. [32]	+	+	N/A	+	N/A	+	+	+	+	+	+
Wiafe et al. [33]	+	+	N/A	+	N/A	+	+	+	+	+	+
Gebreyesus et al. [34]	+	+	N/A	+	N/A	+	+	+	+	+	+
Chalise et al. [35]	+	+	N/A	−	N/A	+	+	+	+	+	+
Shaka and Wondimagegne [36]	+	+	N/A	+	N/A	+	+	+	+	+	+
Ahankari et al. [37]	+	+	N/A	+	N/A	+	+	+	+	+	+
Regasa and Haidar [38]	+	+	N/A	+	N/A	+	+	+	+	+	+
Agrawal et al. [39]	+	+	N/A	+	N/A	+	+	+	+	+	+
Thomas et al. [40]	+	−	N/A	+	N/A	+	+	+	+	+	+
Nelima [41]	+	+	N/A	−	N/A	+	+	+	+	NR	+
Ramzi et al. [42]	+	+	N/A	−	N/A	+	+	+	+	+	+
Kaur et al. [43]	+	+	N/A	+	N/A	+	+	+	+	NR	+
Leenstra et al. [44]	+	+	N/A	+	N/A	+	+	+	+	+	+
El Sahn et al. [45]	+	+	N/A	+	N/A	+	+	+	+	+	+

N/A, not applicable (due to cross-sectional design of study); NR, not reported. +, positive overall score: this overall score is given if criteria 2, 3, 6, and 7 of the QCC and one additional criterion have received a positive score. Ø, neutral overall score: this score is given if more criteria are met than for a negative overall score, but an overall positive score is not reached. −, negative overall score: this score is given if six or more QCC criteria are not met.

## Data Availability

The data supporting this systematic review are from previously reported studies and datasets, which have been cited. The processed data can be obtained from the corresponding author upon reasonable request.
